# How does coping influence impulsivity in patients with remitted bipolar disorder?

**DOI:** 10.1192/j.eurpsy.2021.534

**Published:** 2021-08-13

**Authors:** N. Messedi, E. Mhiri, F. Charfeddine, O. Bouattour, L. Aribi

**Affiliations:** 1 Psychiatry (b), Hedi Chaker University hospital, sfax, Tunisia; 2 Psychiatry, Hedi Chaker University hospital, sfax, Tunisia; 3 Psychiatry (b), Hedi Chaker University hospital, Sfax, Tunisia

**Keywords:** Bipolar, Remitted, Impulsivity, Coping

## Abstract

**Introduction:**

Impulsivity is an important component of the phenomenology of bipolar disorder. Recent studies show that bipolar patients use various strategies to deal with life stressors and with the discomfort related to their disease.

**Objectives:**

To study impulsivity and coping strategies in bipolar patients in remission phase and the factors associated with them.

**Methods:**

A cross-sectional, descriptive and analytical study of 30 patients followed for bipolar disorder, in remission, at the psychiatric outpatient clinic at CHU Hédi Chaker in Sfax. We used a socio-demographic and clinical data sheet, the Ways Of Coping Checklist to assess coping and the Barratt Impulsivity Scale to assess impulsivity.

**Results:**

The average age was 43.77 years, the sex ratio was 0.5. Smoking was found in 30%. Bipolar I disorder was diagnosed in 93% of patients. The mean age of onset was 27.8 years, and the mean duration of illness was 15 years. *Impulsivity was found in 20% of cases and was correlated with the duration of the disease (p = 0.016) and smoking (p = 0.009). *Coping focused on the problem present in 70% of patients, correlated with the duration of the disease (p = 0.032) and coping (p=0.02). *Emotion-centered coping revealed in 20% of patients, correlated with gender (p = 0.037) and cognitive impulsivity (p=0.032). *Coping focused on seeking social support was present in 10% of patients.

**Conclusions:**

Impulsivity is quite frequent in remitted bipolar patients, who mainly used problem-focused coping and a cognitive management of the stressful event. Thus the hypothesis was that impulsivity is core trait of bipolar disorder.

